# Villous Papilloma of the Rectum1Read before the American Proctologic Society, Atlantic City. N. J., June 3-4, 1907.

**Published:** 1907-08

**Authors:** Benjamin Merrill Ricketts

**Affiliations:** Cincinnati, Ohio


### ABSTRACTS.

### Villous Papilloma of the Rectum.[1]

BY BENJAMIN MERRILL RICKETTS, M.D., LL.D., Cincinnati, Ohio

[*Author's Abstract.*]

AFTER mentioning its rarity and disposition to undergo malignant change, the author gives a historical resume beginning with the first case reported by Quain, 1885. Since this time there have been sixty-two cases reported by twenty-one authors, six of which have been Americans, reporting nine cases.

1. Read before the American Proctologic Society, Atlantic City, N. J., June 3-4, 1907.

It occurs most frequently between the ages of thirty-five and sixty, the youngest reported being seventeen years old. There is no known cause, and the growth is supposed to be slow.

Its pathology is dealt with extensively, and symptoms given in detail. Operative methods are intraanal resection and extraanal resection. The former consists of longitudinal and circular resection. Ligature clamp and clamp and cautery. Of these ligature is the most popular. Extraanel resection is accomplished after removing the coccyx.

The technic of each method is given in detail.

Abstracts of thirteen cases is given followed by the report of a personal one, recently twice operated upon.

The first operation by ligature was done Oct. 25, 1906, when a neoplasm giving all the characteristics in history symptoms and appearance of villous papilloma was removed. The second operation, extraanal, was done May 8, 1907, revealing multiple adenocarcinoma. Four inches of the lower rectum were removed; dissolution at the end of eighty hours, probably due to infection.

It is impossible at all times to differentiate villous papilloma from the region of the tumor and radiate downward and outward. reason there is doubt of the two existing at the same time in a given case. Such a condition has not been recorded. The microscopical appearance of the first is that of adenoma with villous characteristics without bloodvessels in the papillæ. That of the second in adenoma undergoing carcinomatous degeneration.

